# High‐Performance Printed Supercapacitors Based on NaOH‐Activated Wood‐Derived Carbon: Optimized Porosity and Long‐Term Stability in Aqueous Electrolytes

**DOI:** 10.1002/smsc.202500540

**Published:** 2026-02-13

**Authors:** Hamed Pourkheirollah, Remuel Isaac M. Vitto, Dāvis Kalniņš, Aleksandrs Volperts, Steffen Thrane Vindt, Līga Grīnberga, Anatolijs Šarakovskis, Gints Kučinskis, Jari Keskinen, Matti Mäntysalo

**Affiliations:** ^1^ Faculty of Information Technology and Communication Sciences Tampere University Tampere 33720 Finland; ^2^ Institute of Solid State Physics University of Latvia Riga LV‐1063 Latvia; ^3^ Laboratory of Lignin Chemistry Latvian State institute of Wood chemistry Riga LV‐1006 Latvia; ^4^ InnoCell ApS Svendborg 5700 Denmark

**Keywords:** activated wood carbon, energy density, energy storage, graphitization, printed supercapacitors, reduced graphene oxide, specific capacitance

## Abstract

The transition to sustainable energy technologies calls for supercapacitors that are not only efficient but also environmentally responsible. In this work, a step towards solving this challenge is taken by applying a low‐temperature NaOH activation strategy to alder‐wood‐derived carbon. Such process generates an amorphous carbon matrix with thinly‐layered sheets of graphene‐like domains, facilitating efficient ion‐electron transport, as revealed through comprehensive material characterizations. Various carbon structures were obtained by adjusting the alkali‐to‐carbon ratio and activation temperature wherein the most effective is 3:1 ratio at 600 °C (AWC 3‐600). Its combined 2393 m^2^ g^−1^ surface area and 85.4% microporosity provides a pore architecture that works exceptionally well with aqueous electrolytes. When integrated into printed supercapacitors, it achieves ≈307 F g^−1^ in NaCl and ≈291 F g^−1^ in K_*x*_H_*y*_PO_4_. Even after 10,000 charge–discharge cycles, the devices retain 95% of their original capacitance, demonstrating long‐term stability. The results of this study highlights the strong interactions between the electrolyte and pore structure, where NaCl benefits from the microporous AWC 3‐600while K_*x*_H_*y*_PO_4_ performs better on the mesoporous structure obtained with an activation process of 4:1 ratio at 700 °C (AWC 4‐700). This study shows that low‐temperature NaOH activation offers an effective way to engineer biomass‐derived carbons with tunable electrochemical behavior.

## Graphical abstract

A low‐temperature NaOH activation strategy tailors alder‐wood‐derived carbons into highly porous, graphene‐like structures for printed supercapacitors. The optimized AWC 3‐600 material delivers high capacitance and excellent cycling stability, outperforming commercial benchmarks. The work highlights the crucial role of electrolyte–pore matching and offers a scalable path toward sustainable, high‐performance energy storage.

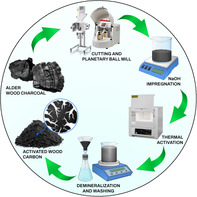

## 1 Introduction

The growing transition from fossil‐based energy systems to renewable energy sources has accelerated global efforts to develop advanced sustainable energy storage system technologies. Due to the intermittent nature of renewable energy sources such as solar and wind, there is a critical need for sustainable, reliable, and efficient energy storage systems to enable their effective integration. Supercapacitors (SCs), also known as electrochemical double‐layer capacitors, have emerged as promising candidates to complement or even replace conventional batteries in various applications. The distinct advantages of SCs,^[^ ^1^ ^]^ such as higher power density, rapid charge–discharge capabilities, extended lifespan, and minimal maintenance requirements, make them appealing for specific applications that require large amounts of power in a short period of time,^[^ ^2^ ^]^ such as portable devices,^[^ ^3^ ^]^ wireless sensor networks,^[^ ^4^ ^]^ self‐powered wearable and flexible electronics,^[^ ^5^ ^]^ and energy‐autonomous Internet of Things systems.^[^ ^6^ ^]^

However, the relatively low energy density of SCs compared to batteries has remained a significant limitation, hindering their widespread application in high‐energy‐demand scenarios. To address this challenge, extensive research efforts have focused on developing novel electrode materials with optimized pore structures, high surface area functionality, and favorable electrical conductivity. Among various material candidates, carbon‐based materials have been widely used in commercial SCs due to their high specific surface area (SSA), excellent electrical conductivity, chemical stability, and cost‐effectiveness.^[^ ^7^ ^]^ In particular, activated carbon (AC) has been employed as the benchmark material for SC electrodes (EDLC type),^[^ ^8^ ^]^ wherein energy storage predominantly occurs through the physical adsorption of electrolyte ions at the electrode–electrolyte interface.^[^ ^9^ ^]^

Within the various synthesis routes, the utilization of biomass‐derived precursors for producing AC has gained growing interest in recent years. This approach not only supports the development of sustainable and environmentally friendly energy storage systems, but also adds value to agricultural and industrial waste materials. A broad range of biomass sources, including coconut shells,^[^ ^10^ ^]^ nutshells (e.g., walnut,^[^ ^11^ ^]^ pistachios^[^ ^12^ ^]^), fruit peels (e.g., banana,^[^ ^13^ ^]^ orange,^[^ ^14^ ^]^ mango^[^ ^15^ ^]^), corn husks,^[^ ^16^ ^]^ sugarcane bagasse,^[^ ^17^, ^18^ ^]^ bamboo,^[^ ^19^ ^]^ algae,^[^ ^20^ ^]^ sawdust,^[^ ^21^ ^]^ and various types of wood,^[^ ^22^ ^]^ has been investigated for the production of AC. These natural precursors possess rich carbon content and an inherent porous structure, which favor the activation process. Depending on the precursors and the activation method, either physical (e.g., steam or CO_2_) or chemical (e.g., KOH, H_3_PO_4_), the resulting AC can exhibit a wide range of electrochemical and textural characteristics.

Among these biomass sources, wood‐based precursors are outstanding because of multiple advantageous properties. These properties include the anisotropic and hierarchical cellular structure of wood, which contributes to the aligned pores and channels at the micro‐, macro‐, and meso‐levels, resulting in the desirable porous and textural features in the final AC.^[^ ^23^ ^]^ The well‐developed hierarchical porosity present in wood‐based AC combines high microporosity—essential for charge storage—with mesopores and macropores, which facilitate ionic diffusion and electrolyte accessibility. Furthermore, wood is abundantly available, cost‐effective, and easy to process. In addition, it possesses a tunable composition and structure—depending on the species and treatment conditions—which makes it feasible to achieve improved electrochemical performance through fine‐tuning. These properties make wood‐based AC a promising candidate for sustainable and scalable energy storage solutions.

In general, wood‐derived AC is commonly referred to as AWC (activated wood carbon). In one of the reported studies, Atika et al.^[^ ^24^ ^]^ developed a SC electrode utilizing lignin‐rich porous KOH‐activated carbon derived from eucalyptus wood and demonstrated that activation at a lower temperature (600 °C) resulted in enhanced performance compared to the conventional 800 °C, owing to the preservation of oxygen‐rich surface functionalities. The resulting PACE‐600 electrode exhibited a specific capacitance of 230 F g^−1^ in 2 M NaCl, good cycling stability over 10 000 cycles, and enabled the fabrication of a 1.6 V symmetric device capable of powering a red LED, achieving an energy density of 41 Wh kg^−1^. In another work, Hamouda et al.^[^ ^25^ ^]^ reported the synthesis of hierarchical porous AWC derived from acacia wood using various chemical activation agents such as KOH, ZnCl_2_, and H_3_PO_4_. The KOH‐activated sample (AWC‐K) exhibited a specific surface area of 1563 m^2^ g^−1^ and achieved a specific capacitance of 224.9 F g^−1^ in 2 M KOH. The AWC‐K//AWC‐K symmetric device also demonstrated an energy density of 23.98 Wh kg^−1^ with excellent cycling stability over 10 000 cycles. Malhotra et al.^[^ ^26^ ^]^ developed AWC by chemically activating two bio‐oil fractions (aerosol and condensed) and bio‐char precursors using KOH. These precursors were derived from the fast pyrolysis of stem wood from pine and spruce. The resulting materials exhibited a hierarchical porous structure and surface areas of 1300–1500 m^2^ g^−1^, along with electrochemical performance achieving specific capacitances of 149–152 F g^−1^ at 50 mA g^−1^. The bio‐oil‐derived AWC electrodes showed minimal metal impurities, offering a clear advantage over bio‐char‐based AWC electrodes, with slightly higher energy storage performance. In another research reported in the literature, Zulamita et al.^[^ ^27^ ^]^ investigated the utilization of Kraft lignin, derived from Kraft pulp, to produce AWC electrodes for SC applications. They used the chemical activation method with three different agents (H_3_PO_4_, K_2_CO_3_, and KOH) at two impregnation ratios (1:1 and 2:1) and a constant activation temperature. The fabricated AWCs showed specific surface areas ranging from 458 to 1515 m^2^ g^−1^. Using an aqueous electrolyte, the electrochemical performance was conducted, demonstrating specific capacitance values between 76 and 236 F g^−1^. The two highest specific capacitances were achieved for the electrodes activated via KOH and K_2_CO_3_, respectively. Furthermore, following the ANOVA analysis,^[^ ^27^ ^]^ the activation agent was found to be the most significant factor influencing the specific surface area, pore volume ratio, and the resulting specific capacitance. The KOH‐treated samples exhibited the best performance, in terms of energy and power density (18.47, and 49.48 W kg^−1^, respectively) and cycling stability over 2000 cycles.

This work encompasses the synthesis of three carbon materials from alder‐wood charcoal through thermal carbonization, followed by refinement to achieve a uniform particle size. Sodium hydroxide (NaOH) is used as the activation agent, providing an alternative to the more common KOH‐based activation methods. NaOH is selected because of its lower cost, wider availability, and potential to produce highly microporous structures. The sorptometry results in this article exhibit that the AWC samples possess significantly higher Brunauer–Emmett–Teller (BET) surface area and micropore volume in comparison to the commercial benchmark Kuraray YP‐80F.

Printed electronics presents a transformative approach for fabricating energy storage devices, accompanied by benefits such as scalability, cost‐efficiency, and design flexibility.^[^ ^28^, ^29^ ^]^ Herein, we report on the successful fabrication of printed SCs utilizing optimized and formulated AWC‐based inks which electrochemically outperform not only conventional commercial materials but also previously reported AWC variants from our own work.^[^ ^30^ ^]^ The high specific capacitance and energy density and better long‐term cycling performance observed in the devices of this work are indicative of the effectiveness of our approach in material engineering and activation methodology.

In the present study, the activation temperature and precursor‐to‐activator agent ratio are carefully optimized to achieve the highest possible electrochemical performance in the fabricated printed SCs. Our findings, when compared with those of the previous article,^[^ ^30^ ^]^ reveal that lowering the activation temperature to 600 °C improves electrochemical performance, likely due to the retention of beneficial oxygen‐rich functional groups. However, elevating the NaOH activation agent ratio is found to diminish the performance, necessitating a delicate balance between porosity development and the preservation of electrochemically active sites. While electrolyte‐pore size matching has been widely reported, our work introduces a NaOH activation strategy for alder‐wood carbon that achieves comparable mesopore/micropore balance at lower activation temperatures, potentially reducing energy cost and enabling environmentally sustainable SC fabrication.

Two aqueous electrolytes, NaCl and K_*x*_H_*y*_PO_4_, are selected for the electrochemical experiments undertaken in this research, due to their environmental friendliness and practical advantages over common alternatives like KOH and Na_2_SO_4_. K_*x*_H_*y*_PO_4_ offers high buffer capacity and safer handling than corrosive KOH. In contrast, NaCl is widely available and can be applied at lower temperatures than Na_2_SO_4_. These choices support the study's aim of developing sustainable and practical SC technologies.

## 2 Experimental Section

### 2.1 Preparation of Activated Wood Carbon

The electrode materials for the printed SCs used in this work were three derivatives of AWC developed by the project partner LSIWC, as well as the commercial AC Kuraray YP‐80F as a benchmark for comparison. The AWC materials were sourced from alder‐wood charcoal and activated through a meticulously engineered process to achieve enhanced porosity and surface area.

To begin with, the charcoal precursor (SIA Fille) was ground in two stages—first using a Retsch SM‐100 cutting mill (1 mm sieve), followed by fine milling with a Fritsch Pulverisette 5/2 planetary mill equipped with zirconia mortars and balls. This procedure enables the reduction of particle size to as small as 5 μm. The finely milled charcoal was subsequently combined with NaOH using alkali‐to‐carbon ratios of 2:1, 3:1, and 4:1.

In the next step, the NaOH‐impregnated samples were thermally activated in a Nabertherm 40 L muffle furnace under an argon atmosphere. This process was carried out at two target temperatures, 600 and 700 °C, for a duration of 1 h. Treatment at these temperatures facilitates the decomposition of volatile materials and induces chemical reactions with surface functional groups, leading to the formation of a highly porous structure with randomly oriented slit‐like pores. Following activation, the carbon was demineralized by boiling in 10% hydrochloric acid (HCl) solution for 2 h, then thoroughly rinsed with deionized (DI) water until a neutral pH was attained. The final step was drying the material overnight at 105 °C. The AWC materials used in this study are AWC 2‐600, 3‐600, and 4‐700 in which the first number indicates the alkali‐to‐carbon ratio, and the second number indicates the activation temperature. For instance, AWC 2‐600 represents the sample in which the alkali‐to‐carbon ratio is 2:1, and the activation temperature is 600 °C.

**Figure 1:**
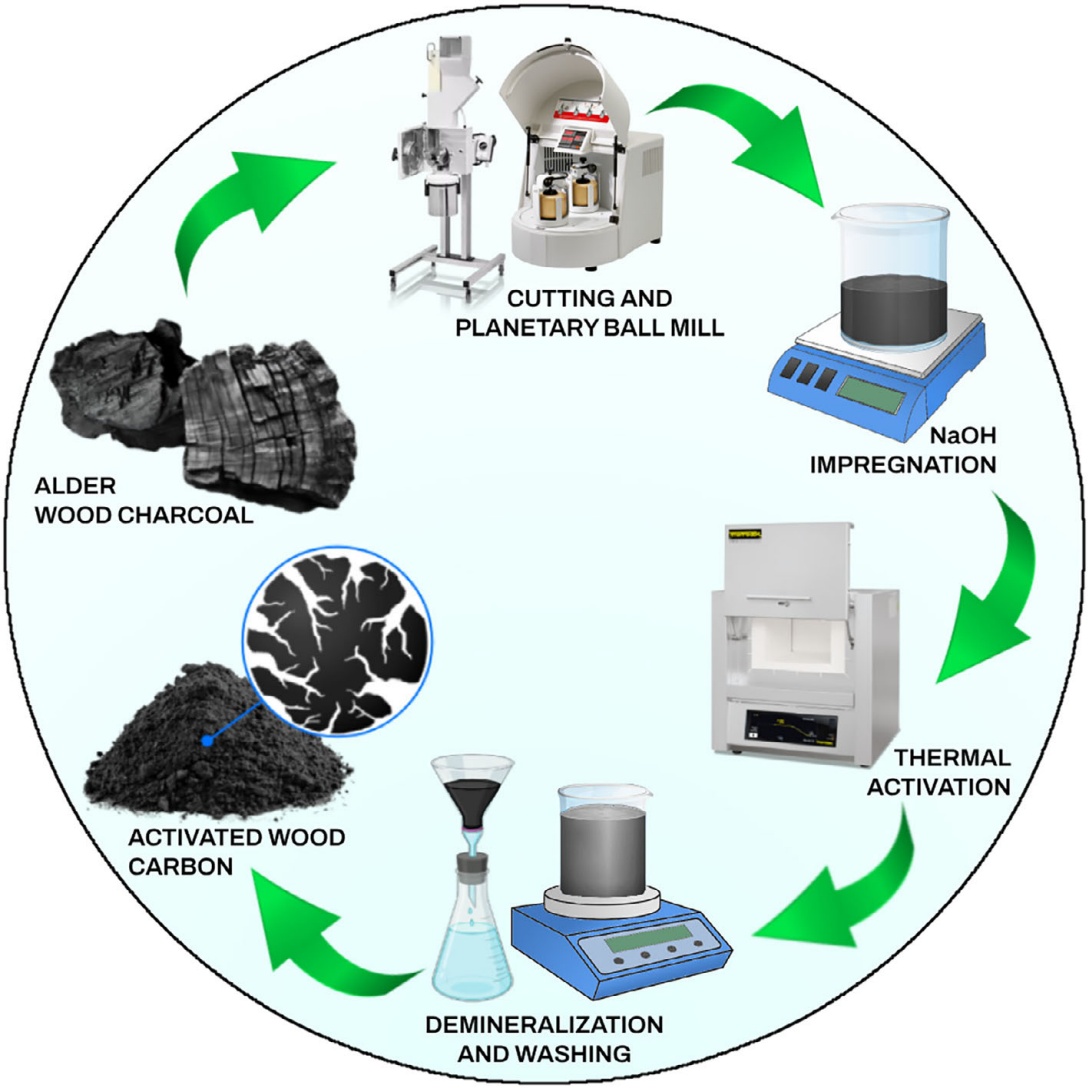
Schematic representation of AWC materials’ activation process steps involving grinding, alkali impregnation, thermal activation, acid washing, and drying.

The activation process ‐including, grinding, alkali impregnation, thermal activation, acid washing, and drying‐ is illustrated schematically in **Figure** **1**. This process makes it feasible to meet the specific performance requirements for SC applications by fine‐tuning and optimization of AC properties.

### 2.2 Materials Characterization

Nitrogen sorptometry analyzes at 77 K were conducted using a Quantachrome NOVA 4200 K instrument to obtain comprehensive volumetric and surface area data, including surface area metrics, pore volume, and pore size distribution. For advanced spectroscopic and structural characterization of AWC samples, Raman spectroscopy was performed using a TriVista CRS Confocal TR777 (Spectroscopy & Imaging GmbH, Germany), X‐ray diffraction (XRD) characterization was carried out using a MiniFlex600 diffractometer (Rigaku Corporation, Japan), and X‐ray photoelectron spectroscopy (XPS) was executed utilizing an ESCALAB 250 Xi+ instrument (Thermo Fisher Scientific, UK) with monochromatized Al Kα radiation for excitation and an X‐ray spot size of 650 × 100 μm. The measurements were performed under high vacuum (≈10^−7^ mbar). Furthermore, in order to examine the surface morphology of the AWC samples, scanning electron microscopy (SEM) analysis was implemented using the SEM Phenom Pro (Phenom‐World, The Netherlands) and Helios 5 UX devices (Thermo Fischer Scientific, USA).

### 2.3 Ink Fabrication

In order to formulate inks from the AC materials, chitosan was employed as the binder. The chitosan solution was prepared by dissolving 1.7 g of chitosan (Sigma‐Aldrich, catalog number 50 494) into a mixture of 67 g DI water and 0.7 g acetic acid (approximately a 1% acetic acid solution). In order to facilitate and accelerate the dissolution process, a magnetic stirrer was used at 50 °C for 1 h. After this step, the chitosan solution, with a total mass of 69 g, was transferred to a zip‐lock bag, into which an additional 20 g of DI water was added. In the next stage, 30.9 g of AC powder was added to the bag, which was then sealed. The mixing of the AC powder with the chitosan solution was initially carried out manually by squeezing and subsequently continued using a small roller for half an hour. The manual squeezing and applying the roller effectively disintegrated any agglomerates within the mixture. However, to achieve the desired low viscosity and smooth fluidic consistency of the ink, additional 6.7 g and 14–18 g of DI water were added to the YP‐80F AC and AWC samples, respectively.

### 2.4 Preparation of Electrolytes

Along with the selection of electrode materials, the choice of electrolyte also plays a key role in determining the overall performance of SCs. Aqueous electrolytes provide notable advantages such as high safety, low cost, environmental compatibility, and high ionic conductivity compared to their organic analogs.^[^ ^31^ ^]^ Moreover, aqueous electrolytes are readily accessible and compatible with a broad range of electrode materials, making them appropriate for diverse SC applications.

In this study, two aqueous electrolytes (NaCl and K_*x*_H_*y*_PO_4_ buffer solutions) have been used in the fabrication of SCs. The sodium chloride (NaCl) electrolyte was prepared by dissolving NaCl into water at a mass ratio of 1:5 (the molarity of the NaCl electrolyte is ≈3.1 M). The potassium phosphate buffer electrolyte (K_*x*_H_*y*_PO_4_) was composed of a mixture of 1 M dipotassium hydrogen phosphate (K_2_HPO_4_) and 1 M monopotassium dihydrogen phosphate (KH_2_PO_4_), formulated to achieve a pH of 7. The pH of this electrolyte was measured using Hanna Instruments HI‐5521, a two‐channel research‐grade benchtop pH/ORP and EC/TDS/Salinity/Resistivity meter.

### 2.5 Fabrication of SCs

The fabrication process of these printed SCs has been previously reported in detail by the authors;^[^ ^30^ ^]^ however, a short summary is provided here. An Al/PET flexible laminate (Pyroll; 9 μm Al, 50 μm PET) was used as the substrate and preheated at 95 °C for 15 min. A graphite current collector (Acheson Electrodag PF‐407C) was coated on the PET side using the laboratory‐scale doctor blade coating technique, with the Al layer acting as a barrier, and dried at 95 °C for 1 h to achieve a 25–35 μm thickness. Then, the AC ink electrode layer was applied and dried overnight at room temperature, forming a 30–40 μm film. In the next step, the 3M 468MP adhesive was applied onto the PET and part of the current collector layer, followed by adding the aqueous electrolyte. Next, a 40 μm Dynacap GT 0.45/40 cellulose paper separator was placed on the electrode and impregnated with electrolyte. Finally, two electrodes (one without the separator) were assembled face‐to‐face and sealed. The final SC dimensions are 50 × 50 × 0.35–0.45 mm.

### 2.6 Characterization of SCs

The key electrical properties of the printed SCs, including capacitance, equivalent series resistance (ESR), and leakage current, were evaluated in accordance with standard IEC 62 391‐1.^[^ ^32^ ^]^ The whole SC device two‐electrode measurements were conducted using a Maccor 4300 workstation (Maccor Inc., USA) over three charge–discharge cycles in the voltage range of 0–1.2 V. Then, the SCs were kept at the constant voltage of 1.2 V for half an hour. Capacitance was calculated during the discharge phase at a constant current between 0.96 and 0.48 V. In order to determine leakage current, the SCs were held at 1.2 V for 1 h. This procedure was repeated at three current levels: 1, 3, and 10 mA. Finally, ESR was derived from the IR drop during the 10 mA discharge step. Besides, the Cyclic voltammetry (CV) and galvanostatic charge–discharge (GCD) curves reported in this paper have also been exported from this characterization method.

To assess the long‐term stability of the printed SCs, charge–discharge cycling was carried out at a constant current of 10 mA up to 1.2 V for 10 000 cycles. Capacitance was monitored and recorded every 100 cycles. All measurements were performed using a Maccor 4300 workstation (Maccor Inc., USA).

## 3 Results and Discussion

### 3.1 Material Characterization

**Table** **1** presents the comprehensive volumetric and surface area sorptometry analysis data for the AWC materials, as well as for YP‐80F benchmark AC. This table includes surface area metrics, pore volume data, and pore size distribution. A variety of analytical techniques, such as MultiPoint BET, DR (Dubinin–Radushkevich) method, and DFT (density functional theory) method, have been employed in this analysis. Further details on these methodologies can be found in.^[^ ^33^, ^34^ ^]^

**Table 1:** Summary of sorptometry data for AC materials.

	Unit	AWC 2‐600	AWC 3‐600	AWC 4‐700	YP‐80F
MultiPoint BET surface area	m^2^ g^−1^	1841	2393	3148	2220
DR method micropore area	m^2^ g^−1^	2266	2535	2759	2063
DR method micropore volume	cc g^−1^	0.8053	0.9010	0.9805	0.7333
DR method micropore width	nm	1.335	1.437	1.471	1.521
micropore/total pore volume	%	99.10	85.40	63.92	70.78
Average pore diameter	nm	1.990	1.974	2.157	2.074
micropore width/ average pore diameter	%	67.09	72.80	68.20	73.33
DFT cumulative surface area	m^2^ g^−1^	1509	1759	1958	1352
DFT cumulative pore volume	cc g^−1^	0.8126	1.055	1.534	1.036

As shown in the table, AWC 2‐600 exhibits the lowest BET surface area (1841 m^2^ g^−1^), whereas AWC 4‐700 shows the highest value (3148 m^2^ g^−1^), much higher than the surface area of benchmark YP‐80F (2220 m^2^ g^−1^). This trend suggests that increasing the activation temperature from 600 to 700 °C, along with raising the NaOH activation agent ratio from 2:1 to 4:1, significantly enhances the surface area of the AC. This increase can be attributed to stronger chemical etching and enhanced pore development during the activation process. At elevated temperatures and with a higher activation agent ratio, the chemical reaction of NaOH with the carbon matrix becomes more effective, leading to the formation of additional pores and enlargement of existing ones. Overall, the accessible surface area is significantly increased. Nonetheless, it should be noted that beyond a certain level of optimization, excessive activation may result in the pore collapse or structural degradation in AC that thereby potentially lower the surface area.

Another important factor to consider in the porosity structure of AC is the contribution of micropore volume (pores with a diameter less than 2 nm) to the total pore volume. To assess this, the ratio of micropore volume to total pore volume is calculated as a percentage for each material in Table 1. As shown in the table, this ratio for YP‐80F is 70.8%, while AWC 2‐600 exhibits the highest micropore volume ratio (99.1%), and AWC 4‐700 shows the lowest value (63.9%). This trend suggests that increasing the activation temperature from 600 to 700 °C and the NaOH activation agent ratio from 2:1 to 4:1 results in the reduction of micropore dominance. This reduction is primarily due to the fact that aggressive activation conditions lead to the widening and transformation of micropores into mesopores (pores with diameters between 2 and 50 nm) and eventually macropores (pores with diameters above 50 nm). In other words, the total pore volume increases, but the relative contribution of the micropores decreases.

Therefore, a tradeoff exists between maximizing the surface area and maintaining a high micropore‐to‐total pore volume ratio. This tradeoff makes the optimization of both activation temperature and agent ratio essential to achieving the best electrochemical performance of the fabricated SC, as will be demonstrated later in the electrochemical results reported in this article.

Additionally, as demonstrated in Table 1, the micropore width of all three AWC materials is lower than that of the benchmark YP‐80F. Regarding average pore diameter, all AWC samples except AWC 4‐700 exhibit smaller average pore sizes. It can also be observed that increasing the activation temperature and alkali‐to‐carbon ratio generally leads to an increase in both micropore width and average pore diameter. This is because higher temperatures and greater chemical activation intensities enhance the etching process, causing micropores to widen and some pore structures to develop into larger mesopores.

Nonetheless, in order to gain the best electrochemical performance, an optimal balance must also be achieved concerning the ratio of micropore width to average pore diameter. According to the data, this ratio increases with activation temperature and agent ratio from AWC 2‐600 to AWC 3‐600 but then decreases for AWC 4‐700. This indicates that beyond a certain point, excessive activation causes pores to grow disproportionately, reducing the relative contribution of micropores. As will be discussed in a subsequent section of this aticle, a higher ratio of micropore width to average pore diameter correlates with improved electrochemical performance in the fabricated SCs.

**Figure 2:**
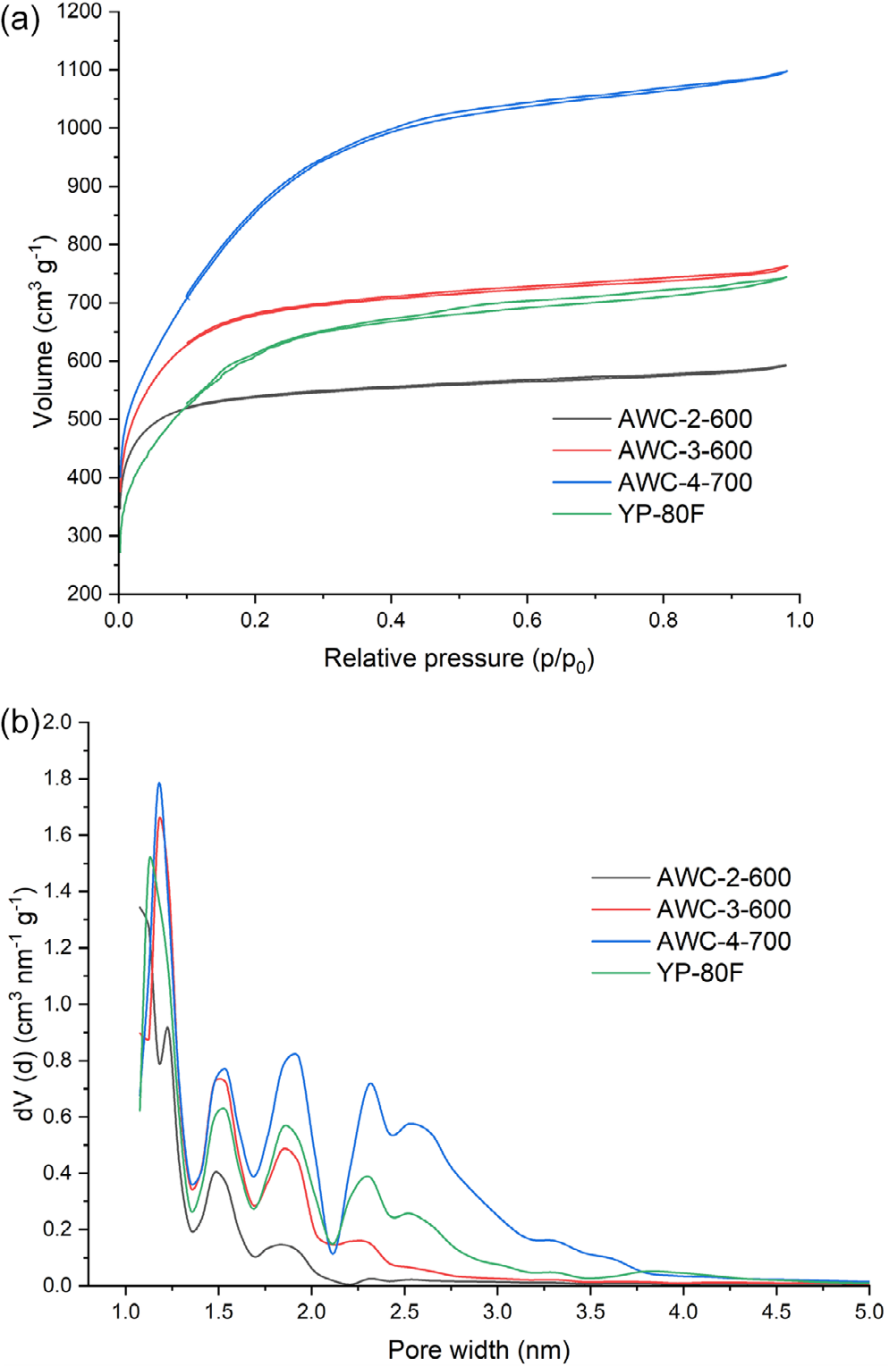
a) Nitrogen sorption isotherms, and b) DFT‐based pore size distributions of AWC and YP‐80F samples.

Nitrogen sorption isotherm analysis at 77 K (**Figure** **2**a) confirms that all samples possess a well‐developed microporous structure. Changes in activation temperature and/or NaOH addition ratio lead to the appearance of hysteresis loops, indicating capillary condensation and the formation of mesopores. Among the samples, AWC 4‐700 (activated at 700 °C with a NaOH ratio of 4) shows the most pronounced mesoporosity, while AWC 2‐600 and 3‐600 (activated at 600 °C) display the least, as supported by pore size distribution data derived from DFT analysis (Figure 2b).

**Figure 3:**
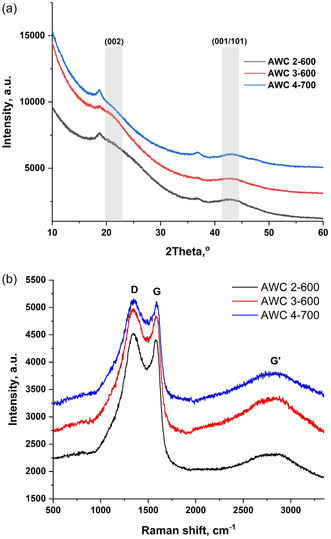
a) XRD patterns, and b) Raman spectra of all AWC samples.

The XRD results and Raman spectra for all three AWC samples can be seen in **Figure** **3**. Figure 3a shows the XRD patterns, demonstrating broad diffraction bands that indicate an amorphous structure composed of randomly oriented reduced graphene oxide (rGO) sheets. Diffraction peaks corresponding to the (002) and (001/101) crystal planes appear at ≈21.35° and 43° (2θ), respectively, and were identified based on JCPDS file no. 75‐1621.^[^ ^35^ ^]^ The appearance of a new peak at ≈18.6° (2θ) may be attributed to the presence of oxygen and moisture trapped between the layers of rGO sheets, or to partially reduced graphene oxide, as discussed by Gupta et al.^[^ ^36^ ^]^

The Raman spectra of all AWC samples (Figure 3b) shows characteristic D (≈1350 cm^−1^) and G (≈1585 cm^−1^) bands, along with a broad G’ (2D) band (≈2700 cm^−1^). For activated carbon, the intensity ratio of the D and G peaks (I(D)/I(G)) and the full width at half maximum (FWHM) of the G peak are commonly used to assess the degree of disorder and heterogeneity in the carbon structure, providing insight into the balance between amorphous and graphitic domains that influence electrochemical performance. The deconvolution of the Raman peaks was performed using Origin software, including initial signal processing, baseline subtraction, and Gaussian functions for peak fitting. The ID/IG ratios and FWHM values are calculated for each sample and are presented in **Table** **2**.

**Table 2:** Raman analysis results of the I(D)/I(G) ratios and FWHM of the G peak.

Sample	I(D)/I(G)	FWHM (G) [cm^−1^]
AWC 2‐600	1 06	184 44
AWC 3‐600	1 36	248 41
AWC 4‐700	1 40	160 40

**Figure 4:**
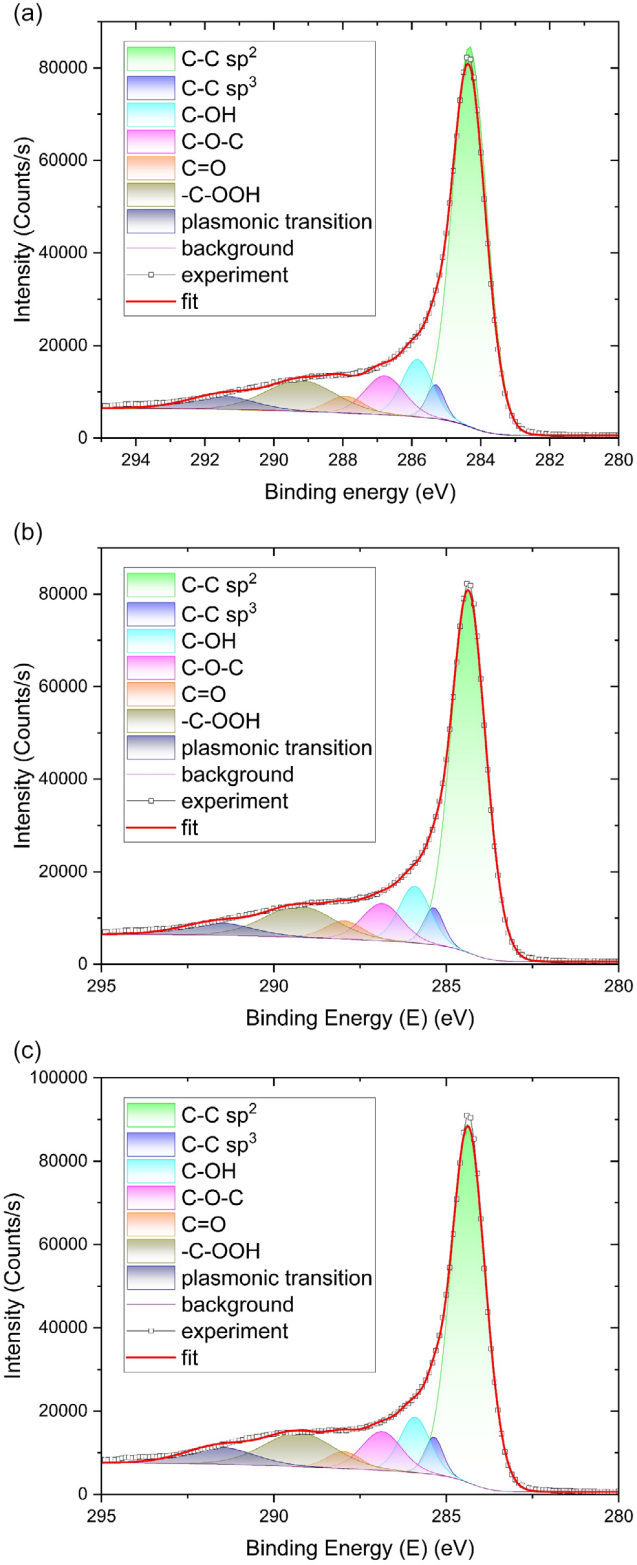
XPS Figures. a) AWC 2‐600. b) AWC 3‐600. c) AWC 4‐700.

The XPS spectra measured for the AWC samples revealed the presence of different carbon‐related functional groups, as shown in **Figure** **4**. The spectra of all the samples are dominated by a peak corresponding to C‐C sp^2^. Additionally, different oxygen‐related functional groups of carbon are observed. The results on binding energy and relative content are summarized in **Table** **3**.

**Table 3:** Binding energies and relative contents of carbon and oxygen functional groups identified by XPS.

	AWC 2‐600		AWC 3‐600		AWC 4‐700		Reference^[^ ^37^ ^]^
	BE, eV	Content, at %	BE, eV	Content, at %	BE, eV	Content, at %	BE, eV
**C sp** ^**2**^	284.3	62.5	284.4	61.6	284.4	60.0	284.1 ± 0.3
**C sp** ^**3**^	285.3	3.0	285.4	3.6	285.4	3.4	285.0 ± 0.2
**C‐OH (hydroxyl)**	285.8	8.5	285.9	8.8	285.9	8.3	285.7 ± 0.2
**C‐O‐C (epoxide)**	286.8	7.5	286.9	7.3	286.9	7.6	286.7 ± 0.2
**C=O (carbonyl)**	287.9	3.0	288.0	3.4	288.0	2.9	288.0 ± 0.3
**‐C‐OOH (carboxyl)**	289.2	10.7	289.3	10.9	289.3	11.8	289.0 ± 0.3
**Plasmonic transition π‐π**	291.6	4.8	291.7	4.5	291.6	6.0	291.5 ± 0.4

**Figure 5:**
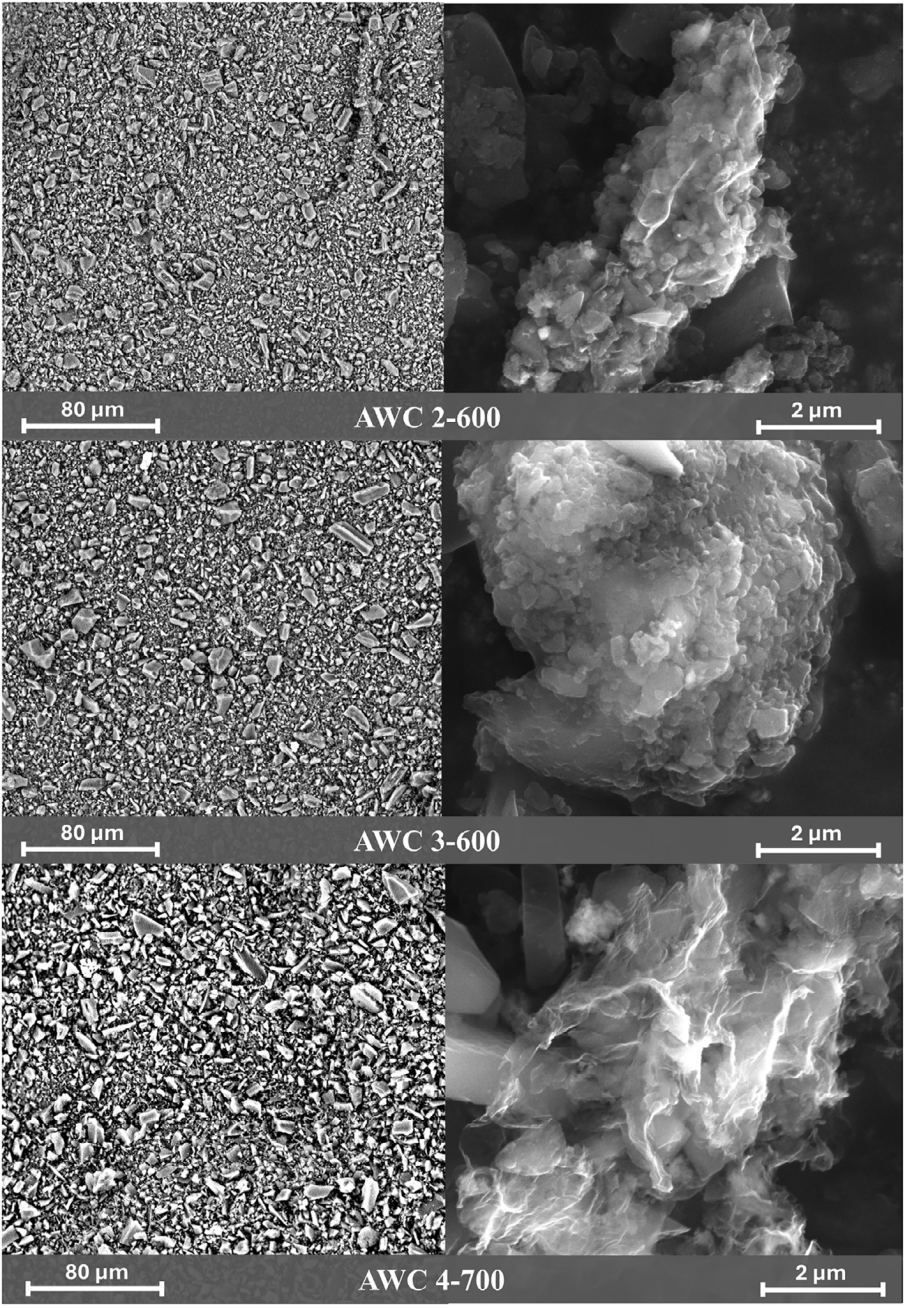
SEM images of all three AWC materials.

The SEM images were taken from the AWC materials before ink formulation. The images (**Figure** **5**) reveal that all three samples are nonhomogeneous, with variations in particle size and morphology. However, AWC‐4‐700 shows larger but more uniform pieces compared to the other two samples. All samples also exhibit graphene‐like sheet structures on some of the grains, indicating the presence of layered carbon domains, that most likely contribute to the G band intensity in Raman spectra. The nonhomogeneous nature explains the broad FWHM, confirming structural heterogeneity. Additionally, the presence of these graphene type structures aligns with the broad peaks around 21.35° and 43° (2θ) in the diffractograms, that are characteristic for the disordered graphitic and reduced graphene oxide structures.

### 3.2 Electrochemical Characterization

**Figure 6:**
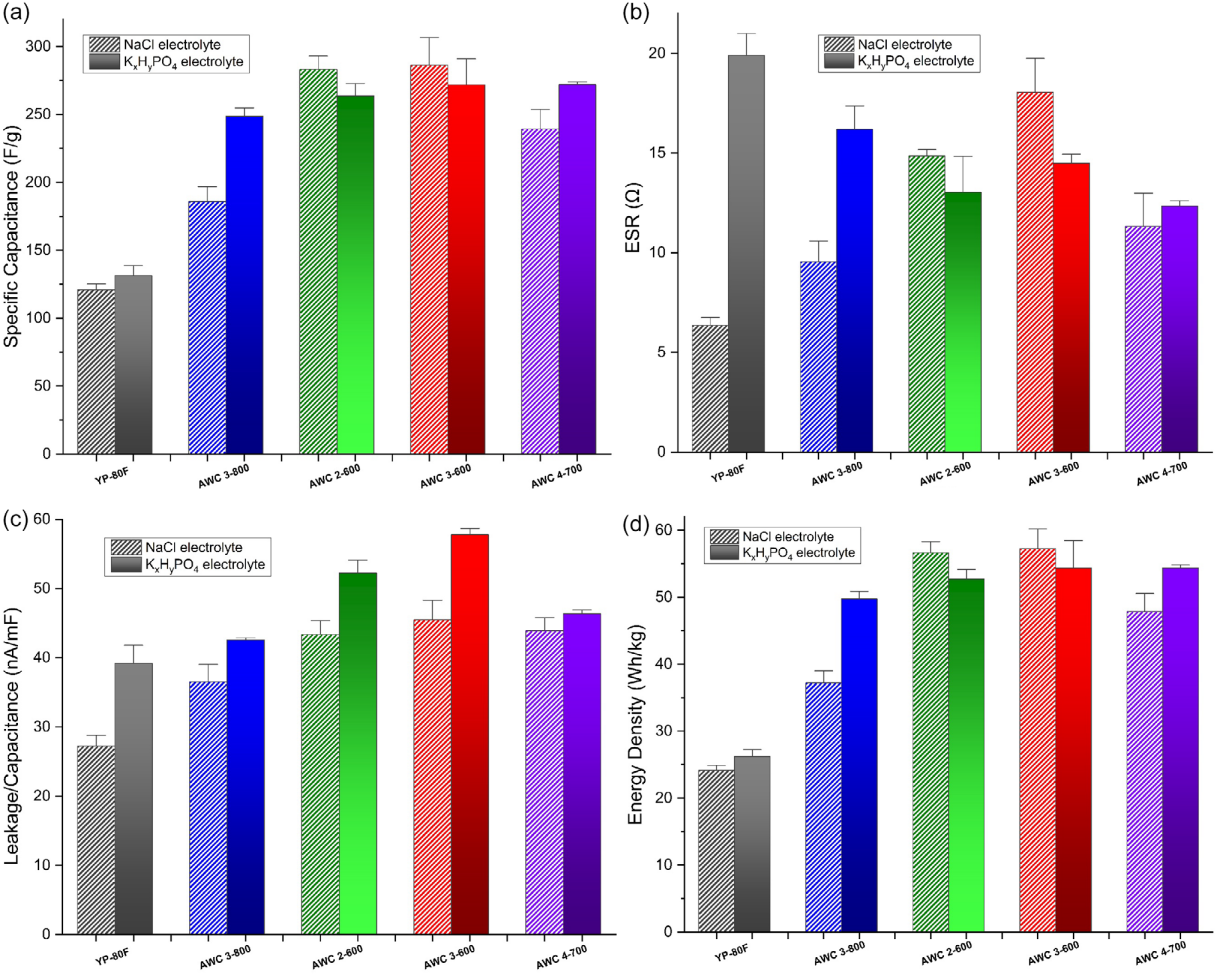
Electrochemical key parameters for a single electrode of printed SCs characterized at 1.2 V, featuring various electrode and electrolyte materials: a) specific capacitance. b) ESR. c) leakage current per capacitance, and d) energy density. Five replicates per type; confidence intervals based on five samples.

To enable a fair and consistent comparison between different AWC materials, the commercial reference AC, and the electrolytes used, five printed SCs are fabricated for each category. The average values of all key parameters are then calculated and compared across these samples. **Figure** **6** presents the various characterization parameters of the fabricated SCs, evaluated at an operating voltage of 1.2 V, which corresponds to the electrochemical stability limit of the two aqueous electrolytes used in this study. To improve the reliability of our results, 90% confidence intervals are included in the bar charts shown in Figure 6. These intervals indicate the range within which the true mean of each parameter is expected to lie with 90% probability. By incorporating confidence intervals, we account for measurement variability and uncertainty, providing a more accurate and robust comparison between different SC types. This approach underscores the precision of the measurements and strengthens the overall credibility of the findings. Besides, in order to enable a comparative analysis and highlight the enhanced electrochemical performance reported in this work, the results for AWC 3‐800 SCs are also included. In our previous publication,^[^ ^30^ ^]^ AWC 3‐800 was identified as the top‐performing material in terms of electrochemical behavior.

#### 3.2.1 Specific Capacitance

The specific capacitance of a single electrode (i.e., the active material on one electrode) in a printed SC is obtained by multiplying the total specific capacitance of the device (measured using the Maccor system) by a factor of four. This is because, in a symmetric SC configuration, both electrodes typically contain nearly equal masses of active material and contribute symmetrically to the overall performance.

Figure 6a demonstrates that the specific capacitance of printed SCs fabricated using AWC materials with optimized activation agent ratios and activation temperatures as presented in this study exceeds that of SCs based on the reference YP‐80 activated carbon, as well as the previously reported AWC 3‐800 SC.^[^ ^30^ ^]^ This improvement is evident for both types of aqueous electrolytes. Particularly, as illustrated in Figure 6a, AWC 3‐600 exhibits the highest specific capacitance among the tested materials for both electrolytes, reaching up to ≈307 F g^−1^ with NaCl and 291 F g^−1^ with K_*x*_H_*y*_PO_4_. These values represent improvements of up to 145% and 56% compared to the benchmark YP‐80F and AWC 3‐800 SCs, respectively, when using NaCl. Similarly, when using K_*x*_H_*y*_PO_4_, the improvements are 110% and 14% relative to YP‐80F and AWC 3‐800 SCs, respectively.

The superior electrochemical performance of AWC 3‐600 SCs compared to the other tested materials can be attributed to a well‐optimized balance between BET surface area and the ratio of micropore volume to total pore volume. As presented in Table 1, this material exhibits a higher specific surface area than the benchmark YP‐80F (2393 vs. 2220 m^2^ g^−1^), while retaining a high proportion of micropores, as 85.4% of the total pore volume is comprised of micropores in this material. This suggests that the increase in surface area does not come at the expense of microporosity, which is crucial for efficient charge storage.

Moreover, AWC 3‐600 shows the highest micropore width to average pore diameter ratio (72.8%) among all the tested samples. This indicates that a significant portion of the pores fall within or near the microporous range (typically <2 nm), suggesting a narrow and favorable pore size distribution. A high micropore width/average pore diameter ratio reflects a structure where the majority of the pores are appropriately sized to maximize ion adsorption and minimize ion transport resistance, which are key features for enhancing double‐layer capacitance. This structural feature ensures that the accessible surface area is not just large, but also efficiently utilized by the electrolyte ions.

These characteristics are particularly well‐matched with the properties of the NaCl electrolyte used in this study (≈3.1 M). The Na^+^ and Cl^−^ ions are relatively small, highly mobile, and fully dissociated in aqueous solution, resulting in high ionic conductivity and rapid ion transport. The dominance of micropores in AWC 3‐600 enables efficient access and confinement of these small ions, which enhances the electrochemical double‐layer capacitance. As a result, AWC 3‐600 paired with NaCl exhibits a higher specific capacitance than when used with K_*x*_H_*y*_PO_4_.

In contrast, K_*x*_H_*y*_PO_4_, used at a lower concentration (1 M), contains larger and more complex phosphate species (e.g., H_2_PO_4_^−^, HPO_4_^2−^). These ions have lower mobility and larger hydrated radii, which limits their accessibility to narrow micropores and slows their diffusion kinetics. Although its well‐preserved pore structure and high surface area allows AWC 3‐600 to maintain strong performance with K_*x*_H_*y*_PO_4_, the restricted ion transport and steric hindrance associated with phosphate ions lead to slightly lower capacitance compared to NaCl.

The variation in specific capacitance performance observed across different AWC materials with NaCl and K_*x*_H_*y*_PO_4_ electrolytes can be attributed to the interplay between the pore structure characteristics and electrolyte ion properties. According to Figure 6a, for AWC 2‐600, the specific capacitance is likewise higher when using NaCl as the electrolyte. This material exhibits an exceptionally high micropore volume to total pore volume ratio (99.1%) and a narrow micropore width of 1.335 nm, which makes it highly compatible with the small, mobile ions of NaCl (Na^+^ and Cl^−^). These small ions can efficiently access the narrow micropores, enhancing double‐layer formation. In addition, the high concentration (3.1 M) and high ionic conductivity of NaCl further support rapid ion diffusion and lower resistance, contributing to improved electrochemical performance. In contrast, the K_*x*_H_*y*_PO_4_ electrolyte contains larger and less mobile ions such as H_2_PO_4_^−^ and HPO_4_^2−^, which experience steric hindrance in the narrow micropores of AWC 2‐600, thereby limiting their access and reducing capacitance.

In contrast, the trend is reversed for AWC 4‐700, with higher specific capacitance obtained when K_*x*_H_*y*_PO_4_ is used as the electrolyte. This material, activated at a higher temperature, possesses the highest BET surface area (3148 m^2^ g^−1^) and total pore volume (1.534 cc g^−1^) among the tested samples, but it has the lowest micropore volume ratio (63.92%) and a larger average pore diameter (2.157 nm). This shift toward a more mesoporous structure reduces the ion‐access resistance for bulkier phosphate ions, making it more suitable for electrolytes like K_*x*_H_*y*_PO_4_. The mesoporous network facilitates the accommodation and diffusion of large ions, allowing more effective charge storage in regions that would otherwise be inaccessible in narrower pore systems. Meanwhile, despite the smaller size of Na^+^ and Cl^−^ ions, the relatively lower microporosity in AWC 4‐700 limits the surface area available for efficient double‐layer formation in NaCl electrolyte, resulting in reduced specific capacitance compared to the K_*x*_H_*y*_PO_4_ case.

These observations highlight the importance of matching pore architecture with electrolyte characteristics. NaCl, with its smaller and faster ions, performs best with highly microporous carbons activated at lower temperature (600 °C) such as AWC 2‐600 and AWC 3‐600, while K_*x*_H_*y*_PO_4_, composed of larger, slower ions, benefits from more open pore carbon structures activated at higher temperatures (700 and 800 °C) like that of AWC 4‐700 and also AWC 3‐800.

#### 3.2.2 ESR

The variation in ESR among the printed SCs fabricated with different AWC materials arises from the combined effects of pore structure, ion transport, and interfacial properties. As shown in Figure 6b, when NaCl is used as the electrolyte, ESR generally increases from YP‐80 F to AWC 3‐800, 2‐600, and 3‐600. This trend can be largely attributed to the high microporosity and narrow pore widths of AWC 2‐600 and 3‐600. While such structures are beneficial for achieving high specific capacitance through efficient double‐layer formation, they restrict ion mobility, particularly under higher current loads which leads to increased ionic resistance and higher ESR. Limited pore connectivity and diffusion bottlenecks in these highly microporous materials further hinder ion transport.

A deviation from this trend is observed for AWC 4‐700, which exhibits a lower ESR than AWC 2‐600 and 3‐600, though still higher than YP‐80F and AWC 3‐800 (Figure 6b). AWC 4‐700 possesses the highest BET surface area (3148 m^2^ g^−1^) and total pore volume (1.534 cc g^−1^), along with a reduced micropore fraction (63.92%) and a larger average pore diameter (2.157 nm). This more open, mesoporous architecture facilitates faster ion diffusion and reduces internal resistance, resulting in improved ESR despite the high surface area.

When K_*x*_H_*y*_PO_4_ is employed as the electrolyte, all AWC‐based SCs exhibit lower ESR values than those based on YP‐80F (Figure 6b). This behavior is attributed to better compatibility between the potassium phosphate buffer and the pore structure and surface chemistry of the AWC materials. The relatively larger ions in K_*x*_H_*y*_PO_4_ benefit from wider and more accessible pores, particularly in AWC 4‐700, which shows the lowest ESR among the AWC samples for this electrolyte. In addition, possible interactions between phosphate ions and residual surface functional groups may reduce interfacial charge‐transfer resistance. The lower electrolyte molarity (1 M) compared to NaCl (3.1 M) may also mitigate ion crowding effects, further supporting efficient ion transport.

Overall, while high microporosity enhances capacitance by maximizing accessible surface area, it can increase ESR due to restricted ion mobility, especially in NaCl electrolyte. In contrast, mesoporous structures such as AWC 4‐700 provide a more favorable balance between surface accessibility and ion transport, particularly for bulkier ions in K_x_H_y_PO_4_, resulting in lower ESR values.

#### 3.2.3 Leakage Current

Leakage current in this paper is evaluated as leakage normalized to capacitance and reported in nA/mF. This metric allows a fair comparison between devices with different capacitance values and is especially useful when comparing materials with varying electrochemical behavior. Lower leakage per capacitance indicates better charge retention and more stable operation, which is important for practical energy storage applications.

As shown in Figure 6c, both electrolytes exhibit the same overall trend. The leakage‐to‐capacitance ratio increases from the benchmark YP‐80F to AWC 3‐800 and rises further for AWC 2‐600 and AWC 3‐600. This increase is associated with the higher porosity and larger microporous surface area of these AWC materials. While microporosity supports higher capacitance, it also introduces more electrochemically active sites, which can promote side reactions and charge redistribution, leading to increased leakage. In addition, very high surface areas may contain defects or residual surface functional groups that further contribute to leakage.

XPS analysis further supports this interpretation. As shown in Figure 4 and summarized in Table 3, all AWC samples display a dominant C–C sp^2^ peak, accompanied by oxygen‐containing functional groups such as hydroxyl (C–OH), carbonyl (C=O), epoxy (C–O–C), and carboxyl (–C–OOH) species. The oxygenated species constitute roughly 30–35 at.% of the total carbon signal, with carboxyl and hydroxyl groups being the most abundant. These functionalities are known to increase the surface polarity and promote side reactions at the electrode–electrolyte interface. In particular, the relatively high content of hydroxyl (C–OH) groups observed in AWC 2‐600 and AWC 3‐600 may contribute to the elevated leakage/capacitance ratio by facilitating slow redox‐like processes and charge redistribution, consistent with the trends observed experimentally.

It is interesting to note that, although AWC 4‐700 has the largest surface area of all the AWC materials, the leakage/capacitance ratio decreases. This divergence implies that variables other than surface area and microporosity also play a role. With a wider pore diameter of 2.157 nm and a more balanced micro‐to‐mesoporous structure (only ≈64%), AWC 4‐700 may restrict localized faradaic reactions or deep ion trapping, which frequently lead to leakage. Its wider range of pore sizes might also allow for less internal potential gradients and more consistent ion transport, which could stabilize charge storage and stop leakage. However, AWC 4‐700's leakage/capacitance is still larger than that of YP‐80F and AWC 3‐800. This is probably because of its large surface area, which continues to contribute to leakage through surface‐related charge dissipation mechanisms.

Furthermore, a noticeable trend is that all printed SCs using the K_*x*_H_*y*_PO_4_ electrolyte exhibit higher leakage/capacitance values compared to their NaCl counterparts. This observation can be attributed to several electrolyte specific properties. The potassium phosphate buffer electrolyte, being composed of multivalent and bulkier ions (e.g., H_2_PO_4_^−^, HPO_4_^2−^), may be more susceptible to slow diffusion and partial trapping in micropores, leading to charge redistribution and time‐dependent potential decay that manifests as leakage. Additionally, phosphate ions may engage in more complex interactions with surface functional groups or residual oxygen‐containing species on the AWC surface, increasing the possibility of slow redox‐like reactions. The lower ionic conductivity of the 1 M K_*x*_H_*y*_PO_4_ electrolyte compared to 3.1 M NaCl may also lead to less efficient charge/discharge cycles and hindered ion removal from deeper pores, contributing to increased retention of stray charges that result in leakage.

All in all, the interplay between electrolyte composition and electrode pore structure significantly influences leakage behavior, and the normalized leakage/capacitance metric proves valuable in capturing these effects systematically.

#### 3.2.4 Energy Density

Regarding energy density, we report the values for the printed SCs based on the single electrode in Wh/kg, calculated using the equation (*C*_s_ × *V* ^2^) / (2 × 3.6), where *C*_s_ is the specific capacitance of the single electrode and *V* is the characterized voltage (1.2 V). As shown in Figure 6d, the printed SCs fabricated with the AWC materials developed in this study exhibit higher energy densities than those using the benchmark YP‐80F AC and the previously reported AWC 3‐800 SC.^[^ ^30^ ^]^ This enhancement is observed for both aqueous electrolytes. AWC 3‐600 delivers the highest energy density among all tested materials, reaching about 61 Wh kg^−1^ with NaCl and 58 Wh kg^−1^ with K_*x*_H_*y*_PO_4_. These values correspond to improvements of up to 145% and 56% over YP‐80F and AWC 3‐800, respectively, in NaCl electrolyte. Similarly, in K_*x*_H_*y*_PO_4_, the enhancements are 110% and 14% compared to YP‐80F and AWC 3‐800, respectively. As discussed earlier in the specific capacitance section, the outstanding electrochemical performance of AWC 3‐600 can be attributed to its well‐balanced combination of high BET surface area, optimal micropore‐to‐total pore volume ratio, and the highest micropore width‐to‐average pore diameter ratio, which together promote efficient ion accessibility and charge storage.

#### 3.2.5 CV and GCD Measurements

**Figure 7:**
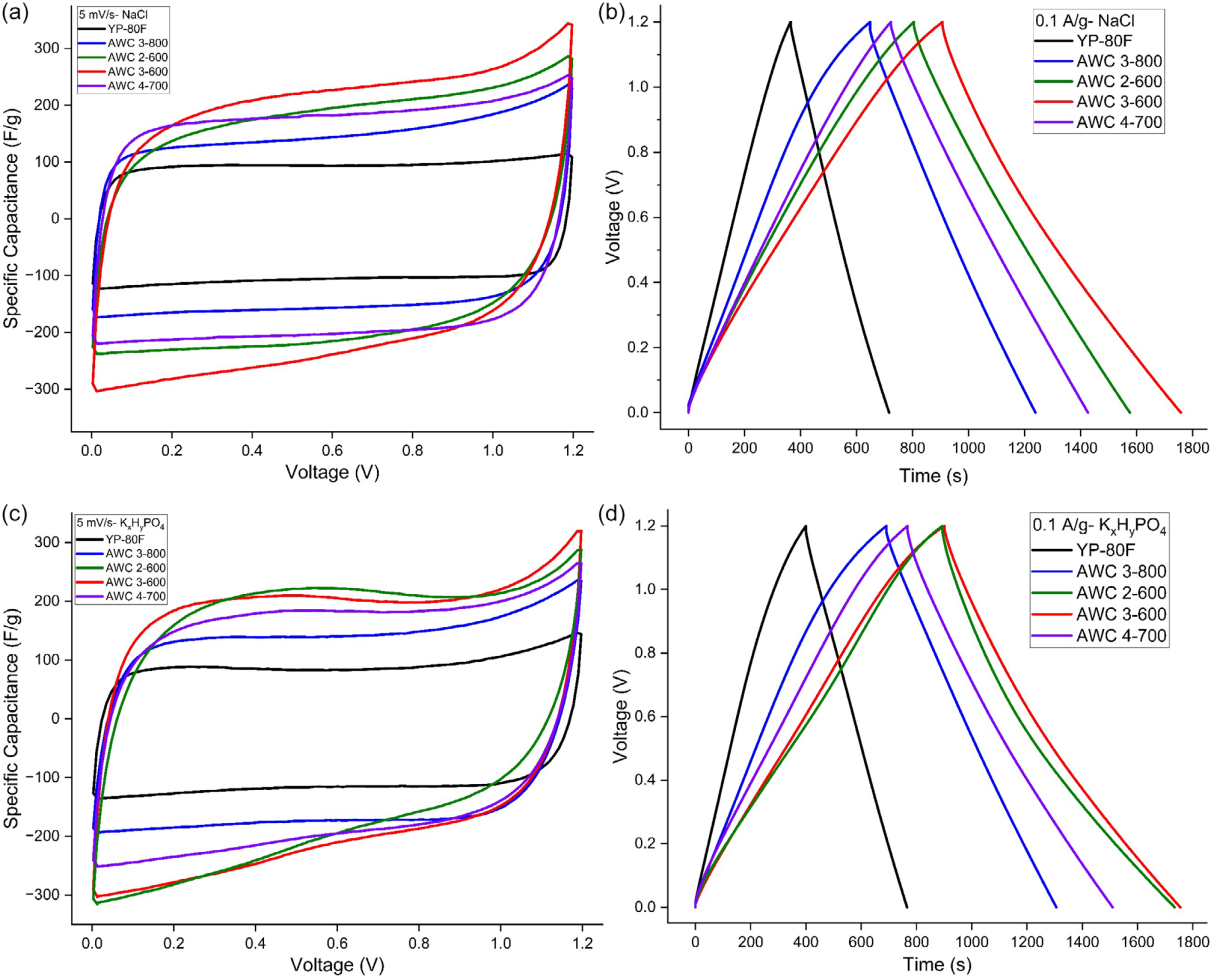
CV (5 mV s^−1^) and GCD (0.1 A g^−1^) curves of printed SCs with YP‐80 F and AWC materials using NaCl and K_*x*_H_*y*_PO_4_ electrolytes at 1.2 V. a, b) NaCl electrolyte SCs. c, d) K_*x*_H_*y*_PO_4_ electrolyte SCs.

CV and GCD tests are also performed to further assess the electrochemical behavior of the printed SCs based on benchmark YP‐80F and different AWC materials, as shown in **Figure** **7**. EDLC behavior can be observed by quasi‐rectangular forms, which are typically seen in the CV curves recorded at a scan rate of 5 mV s^−1^ (Figure 7a, c). However, the CV curves of the AWC‐based SCs show tailing features, which are a departure from perfect rectangularity, especially in the higher voltage range (1.0 to 1.2 V).

There are multiple reasons for this tailing. One possibility is that partial water decomposition and related parasitic reactions resulting from the onset of water electrolysis in aqueous electrolytes close to the upper voltage limit. The surface of the AWC materials may have oxygen‐rich functional groups that could undergo redox reactions at higher voltages and introduce pseudocapacitive behavior, which could be another contributing cause. These effects are more noticeable in materials that have more active sites or a larger density of surface functions, which are the case with AWC materials. According to these findings, surface chemistry and electrolyte stability at higher voltages need to be taken into account in order to fully optimize performance, even though the AWC materials exhibit encouraging capacitive behavior.

Good reversibility and capacitive behavior are confirmed by the symmetric charge and discharge profiles seen in the GCD curves (Figure 7b, d), which were obtained at a current density of 0.1 A g^−1^. The curves are not entirely linear, though. Rather, they display a small amount of curvature, particularly at the first and last phases of the cycles of charging and discharging. This nonlinearity, especially in samples with significant microporosity, can be linked to ion transport restrictions within the carbon electrodes’ porous structure. The observed deviations from linearity are also caused by internal resistance, which is represented in the IR drop, and potential pseudocapacitive contributions from surface groups.

In summary, the CV and GCD profiles support the trends observed in the electrochemical characterization results shown earlier. The tailing in CV curves and the slight curvature in GCD plots provide further insight into the interplay between surface chemistry, pore structure, and electrolyte behavior, particularly at elevated voltages.

### 3.3 Long‐Term Cycling Performance

**Figure 8:**
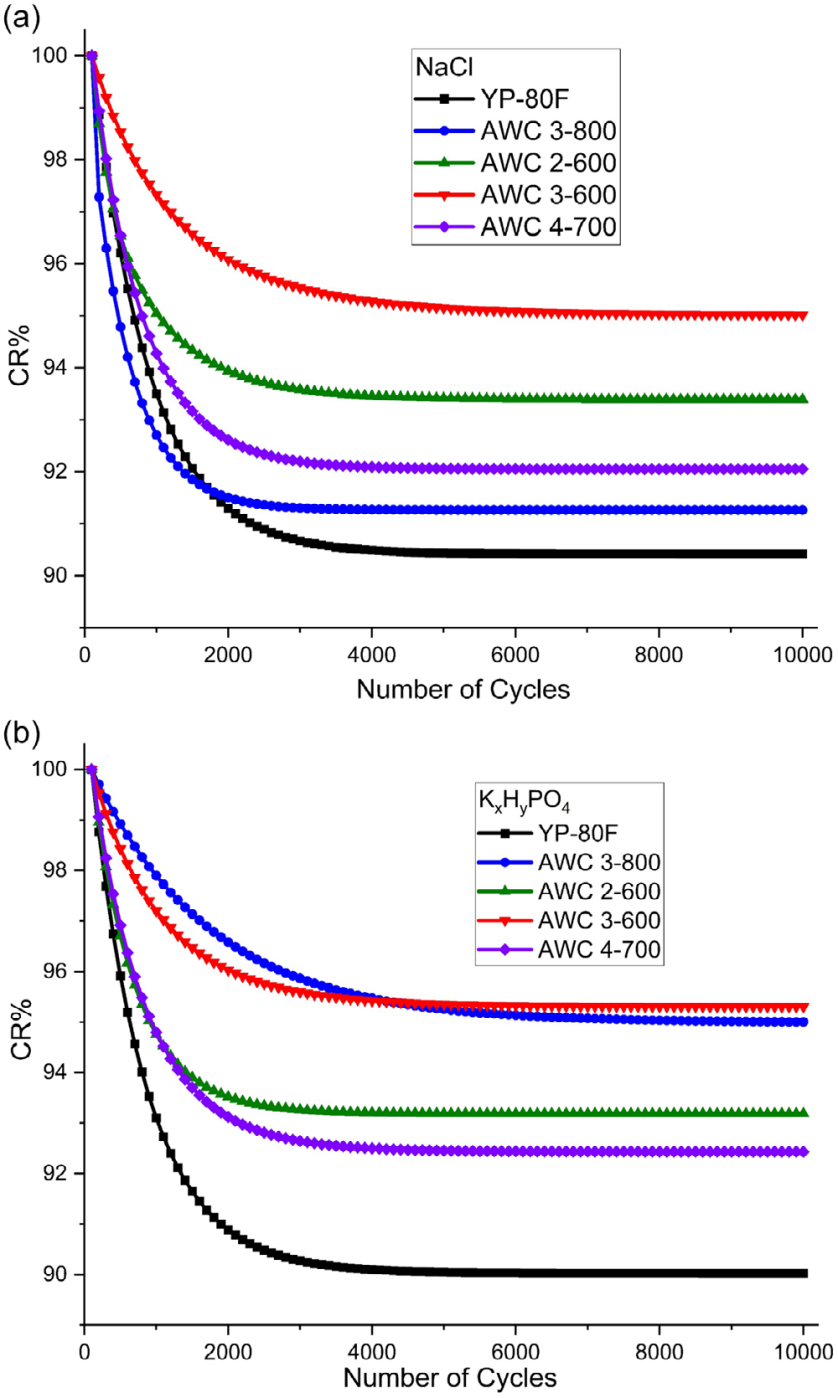
Capacitance retention over 10 000 cycles of printed SCs fabricated using a) NaCl and b) K_*x*_H_*y*_PO_4_ electrolytes.

To evaluate the long‐term cycling performance of printed SCs, 10 000 charge‐discharge cycles were conducted on devices fabricated with NaCl and K_*x*_H_*y*_PO_4_ electrolytes. Capacitance retention (CR% = C_n_/C_0_ × 100) was recorded every 100 cycles, as shown in **Figure** **8**.

Regarding the SCs fabricated using NaCl electrolyte, all AWC‐based SCs surpassed the YP‐80F reference in capacitance retention (Figure 8a). The AWC 3‐600 SC exhibited the most outstanding stability, retaining ≈95% of its initial capacitance after 10 000 cycles, significantly higher than YP‐80 F's ≈90%. Other AWC variants (2‐600, 4‐700, and 3‐800) also performed well, with retentions of ≈93%, 92%, and 91%, respectively. These results underscore the effectiveness of the optimized activation protocol for AWC 3‐600, which likely enhances pore structure robustness and electrode–electrolyte interface stability.

A detailed analysis of the cycling profiles reveals that most capacitance loss occurs within the first 2 000 cycles, after which performance stabilizes. This suggests an initial equilibration phase followed by long‐term stability. Notably, AWC 3‐600 showed the least initial decay and maintained consistent performance beyond 4 000 cycles, demonstrating superior electrochemical durability.

In K_*x*_H_*y*_PO_4_‐based SCs (Figure 8b), AWC 3‐600 and 3‐800 displayed the highest capacitance retention (≈95%), followed by AWC 2‐600 (93%) and 4‐700 (92%), while YP‐80F again lagged at ≈90%. Similar to NaCl, the initial capacitance drop was concentrated in the first 2000 cycles for most materials. However, AWC 3‐600 and 3‐800 exhibited a more gradual decline, stabilizing only after ≈4000 cycles, indicating slower degradation and enhanced structural resilience.

## 4 Summary and Conclusion

In summary, this study demonstrates the successful development of high‐performance printed SCs using AWC materials derived from alder wood. The findings of this paper establish NaOH‐activated wood carbon as a sustainable high‐performance material for printed SCs. Through careful optimization of NaOH activation conditions, we identified AWC 3‐600 (3:1 NaOH ratio, 600 °C activation temperature) as the best‐performing material, combining high surface area (2393 m^2^ g^−1^) with optimal microporosity (85.4% micropore volume). The printed SCs showed exceptional electrochemical performance, with AWC 3‐600 one achieving 307 F g^−1^ specific capacitance and 61 Wh kg^−1^ energy density when using NaCl electrolyte, and 291 F g^−1^ specific capacitance and 58 Wh kg^−1^ energy density when using K_*x*_H_*y*_PO_4_ electrolyte, significantly outperforming commercial YP‐80F. All AWC‐based devices exhibited excellent cycling stability, retaining over 91% capacitance after 10 000 cycles. The study also highlights how activation parameters can be tuned to optimize pore structure for specific electrolyte systems, with NaCl performing best in microporous AWCs while K_*x*_H_*y*_PO_4_ worked better with more mesoporous structures.

Beyond these results, the work underscores the scalability and environmental advantages of using biomass‐derived precursors and simple activation routes to produce high‐quality carbon materials. The water‐based ink formulation and printing process offer an environmentally friendly and potentially cost‐effective pathway for large‐scale fabrication of flexible energy storage devices. Furthermore, the insights gained into the relationship between activation parameters, pore structure, and electrolyte compatibility provide useful guidance for rational materials design in next‐generation sustainable SCs.

As a conclusion, in this work, the successful formulation of AWC‐based inks and their integration into printed devices demonstrate the practical viability of this approach for scalable energy storage solutions. The outcomes of this research provide valuable insights for designing biomass‐derived carbon materials with tailored properties for SC applications, while also contributing to the development of more sustainable energy storage technologies. While this study focuses on material development and device performance trends, electrochemical impedance spectroscopy (EIS) analysis, detailed ink rheology, and device‐level Ragone plots are currently under investigation and will be addressed in future studies.

## Conflict of Interest

The authors declare no conflict of interest.

## Data Availability

Related data and metadata will be openly available in Zenodo at https://doi.org/10.5281/zenodo.15628303.
